# Examination of differences in health indicators between efficient and inefficient countries

**DOI:** 10.12669/pjms.35.1.255

**Published:** 2019

**Authors:** Gozde Yesilaydin

**Affiliations:** Gozde Yesilaydin, PhD. Eskisehir Osmangazi University, Faculty of Health Sciences, Department of Healthcare Management, Eskisehir, Turkey

**Keywords:** Efficiency, Fuzzy Data Envelopment Analysis, Healthcare, Statistical Analysis

## Abstract

**Objective:**

This study determined whether there is a statistically significant difference between efficient and inefficient Organization for Economic Co-operation and Development (OECD) countries in terms of health indicators using fuzzy data envelopment analysis (FDEA).

**Methods:**

In the study, FDEA was performed with three input variables directly affecting health, four environmental factors considered to indirectly affect health, and two output variables. Literature research was used to determine appropriate variables. In FDEA, three different α-cut levels were used. The hypotheses regarding whether there was a statistically significant difference between efficient and inefficient countries in input and output variables were tested for all α-cut levels of upper bound efficiency values.

**Results:**

In terms of health indicators, 17 countries were efficient at α-cut 0 and 0.5. At α-cut 1, 18 countries were efficient. There was only a statistically significant difference between the efficient and inefficient countries in “the number of physicians.”

**Conclusion:**

This study shows the number of physicians was the most important determinant affecting the efficiency of a country’s healthcare system. Inefficient countries had a greater mean for number of physicians. Thus, inefficient countries consume more resources than efficient ones.

## INTRODUCTION

Countries are under obligation to use healthcare resources effectively without compromising on quality.^1^ It is very important for countries to present health services effectively and efficiently and to improve their efficiencies.^2^ Thus, countries have introduced healthcare reforms to improve their performance^3^ and all areas of their operations, review their health policies, and constantly measure their efficiency and productivity. Therefore, the need for a sustainable, efficient, and effective healthcare system is an important issue worldwide.^4^

There are many factors, health indicators, and indicator definitions developed by national and international organizations, reference groups, and academicians that affect the efficiency of health systems.^2^ For example, according to Schulz & Johnson,^5^ variables affecting psycho-socio-somatic health include environment (physical–natural and man-made, sociocultural-political, education, and employment), heredity, behavior (personal habits and nutrition), and healthcare services (community health, promotion, prevention, cure, and rehabilitation). Consequently, economic and social factors play a role in determining the health efficiency of countries.^1^ Moreover, in recent years, health indicators have been elaborated and sub-dimensions have been defined for each indicator with the “Global Reference List of 100 Core Health Indicators” developed by World Health Organization (WHO).^6^ In addition, health-related issues and goals have been included in the United Nations Millennium Development Goals.^7^

Events or situations that occur in real life might be indefinite in various aspects. Imprecise data can also be encountered due to uncertainty.^8^ In real problems, inputs and outputs are often imprecise.^9^ It is also possible to confront incomplete or incorrect data for health indicators in the statistical evaluation of efficiency about healthcare services. The assessment of healthcare efficiency under uncertainty is extremely important for the effectiveness of health reforms. To deal with uncertainty in evaluating the efficiency of health systems, fuzzy data envelopment analysis (FDEA) can be used^3^ to measure efficiency if data are not known precisely^10^ or there is an incomplete, incorrect, or indefinite datum. In FDEA, imprecision is represented by fuzzy sets or fuzzy numbers.^9^ FDEA, which benefits from fuzzy data, states real life situations more realistically than classical data envelopment analysis (DEA).^11^

This study determined whether there is a statistically significant difference between efficient and inefficient OECD countries in terms of health indicators using FDEA.

## METHODS

### Decision Making Units and Variables

In this study, each of the 36 OECD countries was referred to as a decision making unit (DMU) and included in the analysis. Data were obtained from the OECD database^12^ and the World Bank website.^13^ Relevant literature was reviewed in the process of determining variables. The inputs included in the analysis were *“number of physicians (H1),” “number of total hospital beds (H2),” “current expenditure on health (H3),” tobacco consumption % of population aged 15+ who are daily smokers (H4),” “measles immunization % of children immunized (H5),” “CO_2_ emissions (H6),”* and *“school enrollment, secondary (% gross) (H7).”* The two chosen outputs were *“life expectancy total population at birth (H8)”* and *“infant mortality, no minimum threshold of gestation period or birthweight (H9).”*

### Data Analysis

Data from 2015 were used where data availability was highest. If 2015 data was unavailable, the data for the nearest year was used. According to Retzlaff-Roberts, Chang, & Rubin,^14^ slightly older values of some variables can be used for countries when values of related years are unavailable. This adjustment is a common feature of OECD studies and unavoidable in OECD data. Similar to this, Anderson, Hurst, Hussey, & Hughes^15^ also used data from different years for some variables.

The implementation part of the study consisted of two steps. In the first step of the analysis, the health efficiencies of OECD countries were determined using FDEA. For efficiency measurement with FDEA, Wang, Greatbanks, and Yang’s model^16^ was used. The mathematical model of FDEA used in this study is as above.


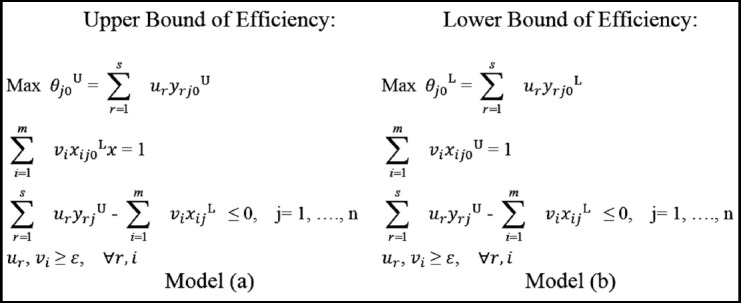


This model was preferred because it is widely used in the literature. Solutions have been made by creating interval data in accordance with the α-cut level approach of Zimmermann.^17^ The α-cut levels used in the study were 0, 0.50, and 1. The data were analyzed using NCSS 10 package program for FDEA.

After efficiency values of countries at different α-cut levels were calculated, the second step determined whether there was a statistically significant difference between efficient and inefficient countries in the selected input and output variables. Therefore, the normality of data was examined. Comparisons between groups were done using independent samples t-test for variables with normal distribution and the Mann-Whitney U test for variables with non-normal distribution. The null hypothesis was rejected at the 5% level.

## RESULTS

Each variable was assessed for a statistically significant difference between efficient and inefficient countries. The data acquired for α = 0, α = 0.5, and α = 1 cut levels of upper bound efficiency values were used. The upper limit was used because the lower limit and upper limit values are the same at the α = 1 level. In addition, there was no efficient country at the lower limits of α=0 and α=0.5. This makes it impossible to compare efficient and inefficient countries with lower boundaries. Statistical data generated by the hypothesis tests are presented in Table-I and Table-II.

**Table-I:** Hypotheses Testing (Independent Samples t–test).

Hypothesis	a = 0^*^			a = 0.50^**^			a = 1^***^		

	t	p	Statistical Decision	t	p	Statistical Decision	t	p	Statistical Decision
H1	-2.529	0.016	Accepted	-2.529	0.016	Accepted	-2.428	0.021	Accepted
H2	-0.335	0.740	Rejected	-0.335	0.740	Rejected	-0.556	0.582	Rejected
H3	0.197	0.845	Rejected	0.197	0.845	Rejected	0.007	0.994	Rejected
H4	-1.089	0.284	Rejected	-1.089	0.284	Rejected	-1.202	0.238	Rejected
H5	-1.433	0.165	Rejected	-1.433	0.165	Rejected	-1.128	0.270	Rejected
H6	-0.233	0.817	Rejected	-0.233	0.817	Rejected	-0.229	0.820	Rejected

p < 0.05 Efficient Countries (n): 17*, 17**, 18*** Inefficient Countries (n): 19*, 19**, 18*** A country was defined as efficient when its efficiency score equaled 1; otherwise, it was defined as inefficient

**Table-II:** Hypotheses Testing (Mann-Whitney U Test).

Hypothesis	a = 0^*^			a = 0.50^**^			a = 1^***^		

	Z	p	Statistical Decision	Z	p	Statistical Decision	Z	p	Statistical Decision
H7	-1.632	0.103	Rejected	-1.632	0.103	Rejected	-1.835	0.066	Rejected
H8	-1.427	0.153	Rejected	-1.427	0.153	Rejected	-1.298	0.194	Rejected
H9	-0.856	0.392	Rejected	-0.856	0.392	Rejected	-1.076	0.282	Rejected

p < 0.05 Efficient Countries (n): 17*, 17**, 18*** Inefficient Countries (n): 19*, 19**, 18*** A country was defined as efficient when its efficiency score equaled 1; otherwise, it was defined as inefficient

Hypothesis 1 was accepted for each of the three alpha cut levels. In other words, there was a statistically significant difference between efficient and inefficient countries in *“the number of physicians” (H1)*. The mean value of inefficient countries was higher than efficient countries. The proper distribution of healthcare resources is very important. Thus, inefficient countries consumed more resources than efficient ones. No other hypotheses were accepted at any alpha level showing that “total number of hospital beds,” “current expenditures on health,” tobacco consumption % of population aged 15+ who are daily smokers,” “measles immunization % of children immunized,” or “CO2 emissions” (Table-I).

Similarly, the hypotheses with non-normal data were not accepted at any alpha level. Therefore, there was not a statistically significant difference between efficient and inefficient countries in “school enrollment,” “life expectancy,” or “infant mortality” (Table-II).

## DISCUSSION

The healthcare system has a major impact on society, just as society impacts healthcare.^5^ Health is an open system interacting with many fields including social and cultural life, economy, politics, technology, and education. Thus, it might be misleading to address health by itself and specify only health-related variables. According to Varabyova & Müller,^4^ although socio economic and lifestyle factors were not direct measures of healthcare inputs, these factors might have an influence on the attainable production set. On the other hand, it is also important to examine environmental variables for determining efficiencies.^1^ Based on this opinion, environmental variables (external variables) such as smoking, immunization, air pollution, and education were included in this study.

Especially in recent years, international comparisons of health system efficiencies have attracted the attention of health policy makers.^18^ In this respect, it is important to evaluate, improve, and analyze the efficiencies of health-related activities of countries at the international level.^1^ Therefore, in this study, it was preferred to evaluate the health efficiency of OECD countries using FDEA.

DEA, which is a relative performance measurement tool,^19^ is widely used in assessing health efficiency. However, the studies that use FDEA are more limited.^3^,^20^-^22^ Most studies using FDEA in healthcare were carried out in micro form at the healthcare institution level such as hospital or clinical units. In the study by Aksoy,^23^ classic DEA and FDEA were used to find health efficiencies of G-20 countries. A study by Yesilaydın and Alptekin^24^ determined health efficiencies of OECD countries by using fuzzy data envelopment analysis. But to the best of the author’s knowledge, no studies using FDEA have been tested significant differences between the efficient and inefficient status of OECD countries using statistical methods.

The hypotheses about whether there is a statistically significant difference between the efficient and inefficient status of countries in input and output variables, which constitute the main purpose of the study, were tested by statistical methods. According to the results, a significant difference was found between efficient and inefficient countries only in “the number of physicians” at all three α-cut levels. Similarly, in the study of Bal & Bilge,^25^ there was a statistically significant difference between efficient and inefficient hospitals in number of physicians and the mean of inefficient hospitals was higher than efficient ones. Unlike the results here, Ravangard et al. found a positive but insignificant relationship between the number of physicians per thousand and health system efficiency.^2^ Varabyova & Muller compared the efficiency of hospitals using the unbalanced panel data from OECD countries during 2000-2009 using similar input, output, and environmental variables as the current study.^4^ However, Varabyova & Muller did not examine any differences between the variables and the efficiency status, as in the current study. A study by Samut and Cafri^1^ investigated the determinants affecting hospitals’ efficiencies across 29 OECD countries using DEA. According to the results, there is a negative significant relationship between health spending and efficiency. Similarly, Ravangard et al.^2^ found a significant positive relationship between GDP per capita and health system efficiency. However, in the current study, there was not a statistically significant difference between efficient and inefficient countries in current expenditure on health.

This study not only contributes to the literature but also provides guidance to health managers, planners, policy makers, decision makers, and academicians who are interested in the measurement of health efficiencies and making cross-country comparisons in healthcare. It has provided evidence of significant differences in countries efficiencies and the important variable for further focus. Future studies should analyze different environmental variables to determine countries’ health efficiencies.
